# Association between dectin-1 gene single nucleotide polymorphisms and fungal infection: a systemic review and meta-analysis

**DOI:** 10.1042/BSR20191519

**Published:** 2019-11-13

**Authors:** Peiru Zhou, Yufei Xie, Zhimin Yan, Xiaosong Liu, Hong Hua

**Affiliations:** Department of Oral Medicine, Peking University School and Hospital of Stomatology, Beijing, China

**Keywords:** dectin-1, fungal infection, single nucleotide polymorphism

## Abstract

**Objectives:** To investigate the association between dectin-1 gene single nucleotide polymorphisms (SNPs) and susceptibility to fungal infection (FI). **Methods:** Databases were searched electronically and manually to identify case–control studies concerning dectin-1 SNPs and FI, which were published up to 12 November 2018. The Newcastle–Ottawa Quality Assessment Scale was used to determine the study quality and bias. The SNP frequencies of the *B* (the variant or minor allele) and *A* (the wild or major allele) alleles of the dectin-1 gene in both cases and controls were analyzed with regard to FI susceptibility. **Results:** Eight high-quality studies were included in the review. Systemic review of the included studies demonstrated that dectin-1 SNPs rs3901533 and rs7309123 might be associated with susceptibility to invasive pulmonary aspergillosis infection; moreover, rs16910527 SNP can possibly increase the susceptibility to oropharyngeal candidiasis in HIV-positive patients. The meta-analysis identified significant associations between dectin-1 SNPs and overall FI risk in the homozygote model (pooled odds ratio (OR) 1.77, *P*=0.04). When classified by subtypes, significant associations were also found for deep FI in the homozygote model (pooled OR 2.46, *P*=0.01) and the recessive model (pooled OR 2.85, *P*=0.002). There appeared to be no significant association between dectin-1 SNPs and superficial FI. **Conclusion:** Systemic review of the included studies suggested that dectin-1 SNPs rs3901533, rs7309123, and rs16910527 might play a role in FI susceptibility. The meta-analysis provided convincing evidence that dectin-1 SNPs might have an important role in FI susceptibility, especially for deep FI.

## Introduction

In nature, there are over 100000 species of fungi, of which 300 species cause diseases in humans and animals [1]. Fungal infection (FI), caused by true or opportunistic fungal pathogens, [2] is an emerging and severe medical concern. FI varies from superficial to deep or systemic types. Superficial FI is a common infectious disease that includes superficial skin infections, like ringworm and nail infections, as well as superficial mucosal infections, such as oral and vaginal thrush. Deep or systemic FI is less common, but can affect any organ when host immunity is compromised or the integrity of the host surface is disrupted, causing serious illness, such as fungal pneumonia or aspergillosis [3].

The risk of invasive FI in healthy individuals is low because of their functioning immune system. However, it was hypothesized recently that host genetic factors might be one of the components determining immunity and thus the susceptibility to FI [4]. Therefore, recognizing genetic risk factors early may lead to the development of individualized management strategies and result in early prevention and better treatments for patients with FI.

The host innate immune system can recognize and eliminate microbial pathogens and provides the first barrier against FI. Dectin-1 (also known as C-type lectin domain family 7 member A (CLEC7A)) is mainly expressed on dendritic cell and macrophage surfaces and is one of the C-type lectin family of transmembrane proteins [5]. β-glycans are major fungal cell wall components that are recognized by dectin-1 via a carbohydrate recognition domain in its extracellular region. Signals are then transduced via dectin-1’s cytoplasmic domain immunoreceptor tyrosine-based activation motif (ITAM). When the ITAM binds its ligand, spleen tyrosine kinase is recruited, activating the caspase recruitment domain family member 9 (CARD9)–nuclear factor κB (NF-κB) axis, which then activates various genes, especially those encoding pro-inflammatory cytokines [6].

Dectin-1 can recognize fungal β-glucans, resulting in the production of soluble mediators and phagocytosis to clear the fungal pathogens. Dectin-1 can also modulate the adaptive immune system via Th1 and Th17 [7]. Thus, both innate and adaptive immune systems are influenced by dectin-1 and therefore, its coding gene (*CLEC7A*).

Polymorphisms in human pattern recognition receptors (PRRs) have been identified as potential predictive factors of infection in susceptible hosts [8]. A functional single nucleotide polymorphism (SNP) in human *CLEC7A* (Y238X, rs16910526) that generates a premature stop codon, leading to a protein lacking the final ten amino acids of the carbohydrate-recognition domain, resulted in decreased expression of the Dectin-1 receptor on immune cell surfaces [9]. The *CLEC7A* intronic SNPs rs3901533 and rs7309123 are associated with susceptibility to invasive pulmonary disease in patients with hematologic diseases; however, the detailed mechanism remains unclear [10].

To clarify this matter, in the present study, a systematic review and meta-analysis were performed to determine whether *CLEC7A* polymorphisms enhance the risk of FI. We identified case–control studies in the literature to investigate the association of the variant allele *B* with the occurrence of FI.

## Materials and methods

This review was performed following the MOOSE guidelines (Meta-analysis Of Observational Studies in Epidemiology) [11].

### Database search strategies

The eligibility criteria comprised case–control studies dealing with the risk of FI and dentin-1 (*CLEC7A*) SNPs. To identify eligible studies, we searched the following databases up to 12 November 2018: Web of Science, PubMed, Embase, Ovid, Cochrane Library, WHO International Clinical Trials Registry Platform (ICTRP), ScienceDirect, WANFANG DATA, VIP INFORMATION, China National Knowledge Infrastructure (CNKI), and Chinese BioMedical Literature Database (CBM). We also manually searched the reference lists of the articles. The searches used the following terms: (‘fungal infection’ OR ‘mycosis’) AND (‘dectin-1’ OR ‘C-type lectin domain family 7 member A’ OR ‘CLEC7A’) AND (‘single nucleotide polymorphism’ OR ‘gene polymorphism’ OR ‘genetic polymorphism’ OR ‘genetic variation’). Two independent investigators conducted the searches (Peiru Zhou and Yufei Xie).

### The diagnostic criteria for FI

Briefly, superficial FI was diagnosed if the infection was caused by a pathogenic fungus and was limited to nails, hair, mucosa, and epidermis. Deep or systemic FI was diagnosed if the infection involved subcutaneous and deep tissue, organs, or even caused disseminated FI [3].

### The inclusion and exclusion criteria

Any case–control study related to FI and dectin-1 gene polymorphisms was considered as eligible for inclusion if it met the following criteria: (i) the outcomes of interest were superficial and deep FI, and the diagnosis of the specific FI diseases was defined according to internationally recognized standards; (ii) there were at least two comparison groups, for example, deep (systemic) or superficial FI versus control groups (subjects with no indication of FI); and (iii) the dectin-1 gene SNPs were determined, frequencies of alleles *B* (*B* represents the variant or minor allele) and *A* (*A* represents the wild-type or major allele) were assessed in both cases and controls. The exclusion criteria were: (i) publication in a language other than English or Chinese and (ii) insufficient information for data extraction.

### Study selection and quality assessment

Two reviewers scanned the titles and abstracts of the identified studies. Full-text articles were read or obtained when no clear judgments could be made by examining the titles or abstracts. Disagreements between the two reviewers were resolved by discussion or by including a third or fourth reviewer (Xiaosong Liu and Hong Hua).

Two reviewers independently carried out the data extraction and arrangement. Data extracted comprised: the authors, country of origin, ethnicity, year of publication, FI type, disease, average age of patients and controls, sample size, SNPs, and genotype frequencies of cases and controls. At data extraction, inter-reviewer reliability was calculated using κ scores. Disagreements were resolved by discussion or by involving a third or fourth reviewer.

The Newcastle–Ottawa Quality Assessment Scale was used to assess the study quality and bias [12]. The scale involves eight scoring items that are assessed from three perspectives: study group selection; group comparability; and whether either the exposure or the outcome of interest for a case–control study is listed in the scale. Each study can receive a maximum of eight stars.

### Statistical analysis

Stata 14.0 (Stata Corporation, College Station, TX, U.S.A.) was used to perform the statistical analyses, together with Review Manager (RevMan) 5.3 (The Nordic Cochrane Centre, The Cochrane Collaboration, Copenhagen, Denmark) and SPSS 17.0 (IBM Corp., Armonk, NY U.S.A.) software. Subtype analyses were undertaken for deep or superficial FI. The estimates of the SNPs effect were plotted on a Forest plot after calculating the odds ratios (ORs) with the 95% confidence interval (CI). The risk was assessed using the allele model (*B vs. A*), the homozygous model (*BB vs. AA*), the heterogeneity model (*AB vs. AA*), the dominant model (*BB* + *AB vs. AA*), and the recessive model (*BB vs. AB* + *AA*). To evaluate the heterogeneity of the studies, we performed an *I^2^* test, which ranged from 0 to 100%; modest and high heterogeneity were determined using *I^2^* cut-off values of 25 and 50% [13]. In the absence of significant heterogeneity (*P*≥0.05), a fixed-effects model was used to calculate the combined OR, otherwise (*P*<0.05), we used a random-effects model. To identify the underlying publication bias, a sensitivity analysis using funnel plots was performed.

## Results

### Characteristics of the studies included in the meta-analysis

**Figure 1:**
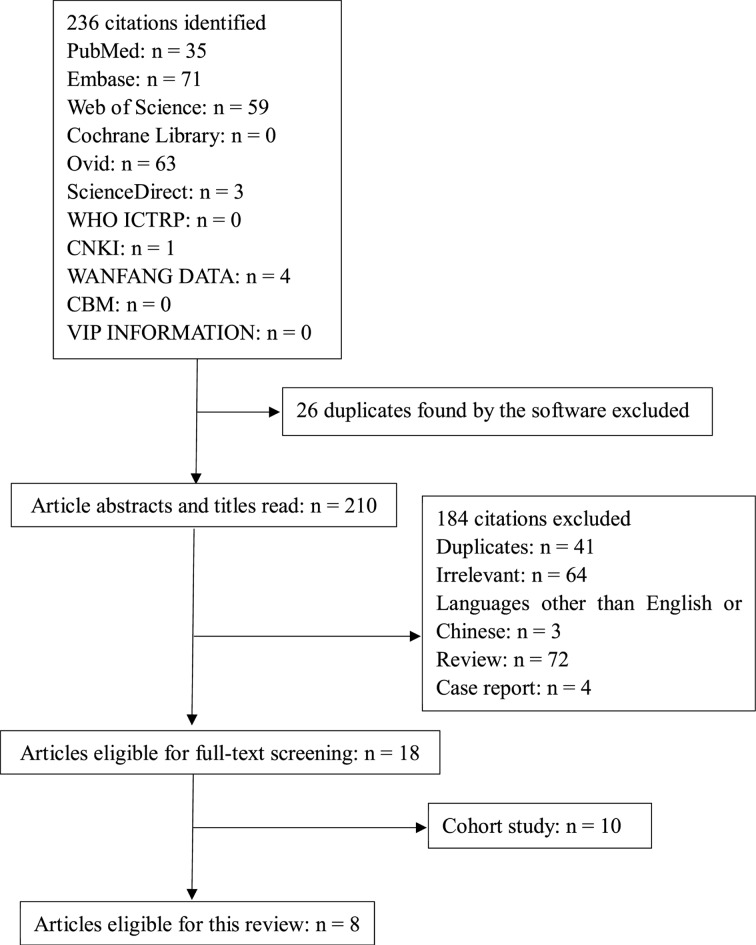
Systematic review flow diagram

Initially, we identified 236 publications, among which 26 were duplicates or did not meet the inclusion criteria, which were discarded. Ultimately, the meta-analysis included eight studies (Figure 1). Among them, four were conducted in the Netherlands, and the rest (one study each) were conducted in China, Spain, Turkey, and the United Kingdom. The eight reports involved a total of 2109 patients with FI, of which four involved 1074 patients with deep FI and the other four involved 1035 patients with superficial FI (Table 1). Superficial FI was reported as fungal keratitis, recurrent vulvovaginal candidiasis, and oropharyngeal candidiasis [14–17]. Deep FI was reported as invasive pulmonary aspergillosis, candidemia, and other invasive FIs [10,18–20].

**Table 1:** Characteristics of the case–control studies included in the systemic review

Study	Country	Ethnicity	FI type	Disease	Average age of		Sample size cases/ controls	SNPs	Number of genotypes (cases/controls)		
					Cases	Controls			*AA*	*AB*	*BB*
Qu, 2015	China	Chinese	Superficial	Fungal keratitis	54.61 ± 10.72	54.61 ± 11.67	109/220	rs17206002	87/184	22/36	0/0
								rs3901533	2/5	42/82	65/133
								rs11053613	82/169	27/51	0/0
								rs3901532	2/6	41/81	66/133
Sainz, 2012	Spain	Caucasian	Deep	Invasive pulmonary aspergillosis infection	48.98 (16-76)	50.95 (16-78)	57/125	rs3901533	35/77	14/43	8/5
								rs7309123	23/49	21/66	13/10
Rosentul, 2014	Netherlands	Western-European	Superficial	Recurrent vulvovaginal candidiasis	NA	NA	119/263	rs16910526	100/219	19/44	0/0
Rosentul, 2011	Netherlands	African-American	Deep	Candidemia	55.9	57.8	93/88	rs16910526	90/84	3/4	0/0
		Caucasian					238/263	rs16910526	200/219	37/44	1/0
Plantinga, 2010	Netherlands	Tanzanian	Superficial	Oropharyngeal candidiasis	35 (18–61)	35 (18–63)	116/108	rs16910526	115/106	2/2	0/0
								rs16910527	108/94	8/12	1/2
Usluogullari, 2014	Turkey	NA	Superficial	Recurrent vulvovaginal candidiasis	29.06 ± 7.3	31.41 ± 6.2	50/50	rs16910526	44/42	6/7	0/1
Chai, 2010	Netherlands	Dutch-Flemish	Deep	Invasive aspergillosis	47 (40–57)	48 (40–56)	71/108	rs16910526	58/96	13/12	0/0
Ceesay, 2016	United Kingdom	White Europeans	Deep	Invasive fungal disease	NA	NA	14/17	rs16910526	13/14	1/1	0/2

Furthermore, we retained records of the reasons for trial exclusion, any disagreements between the two reviewers, and the comments from third or fourth reviewer. At the data extraction stage, inter-reviewer reliability was assessed using the κ score, which was 0.66, suggesting moderate agreement between the reviewers. The study quality assessment is presented graphically by the stars shown in Table 2. All studies were considered to be of high quality (six or more stars for each study).

**Table 2:** Quality assessment of the included case–control studies (using the Newcastle–Ottawa Quality Assessment Scale)

Study	Selection				Comparability	Exposure			Number of stars (score)
	Is the case definition adequate?	Representativeness of the cases	Selection of controls	Definition of controls	Comparability of cases and controls on the basis of the design or analysis	Ascertainment of exposure	Same method of ascertainment for cases and controls	Non-response rate	
Qu, 2015	*	*	*	*	*	/	*	*	7
Sainz, 2012	*	*	*	*	*	/	*	*	7
Rosentul, 2014	*	*	*	*	*	/	*	*	7
Rosentul, 2011	*	*	*	*	*	/	*	/	6
Plantinga, 2010	*	*	*	*	*	/	*	*	7
Usluogullari, 2014	*	*	*	*	*	/	*	*	7
Chai, 2010	*	*	*	*	*	/	*	*	7
Ceesay, 2016	*	*	*	*	/	*	*	/	6

Identify ‘high’ quality choices with a ‘star (*)’. A maximum of one ‘star’ for each item within the ‘Selection’ and ‘Exposure/Outcome’ categories; maximum of two ‘stars’ for ‘Comparability’.

### Pooled effects for *CLEC7A* SNPs as a whole and FI

The meta-analysis included eight studies. One trial [14] assessed four *CLEC7A* SNPs, including rs17206002, rs3901533, rs11053613, and rs3901532 in the same study population. The other two studies [10,16] each assessed two different SNPs of *CLEC7A* (rs3901533 and rs7309123, rs16910526 and rs16910527, respectively) in one study population. Another study [19] compared one SNP of *CLEC7A* (rs16910526) in two populations of different ethnicities (African-American and Caucasian). In this meta-analysis, we separated these studies into subgroups to improve the analysis. Therefore, 14 subgroups in all were analyzed.

**Figure 2:**
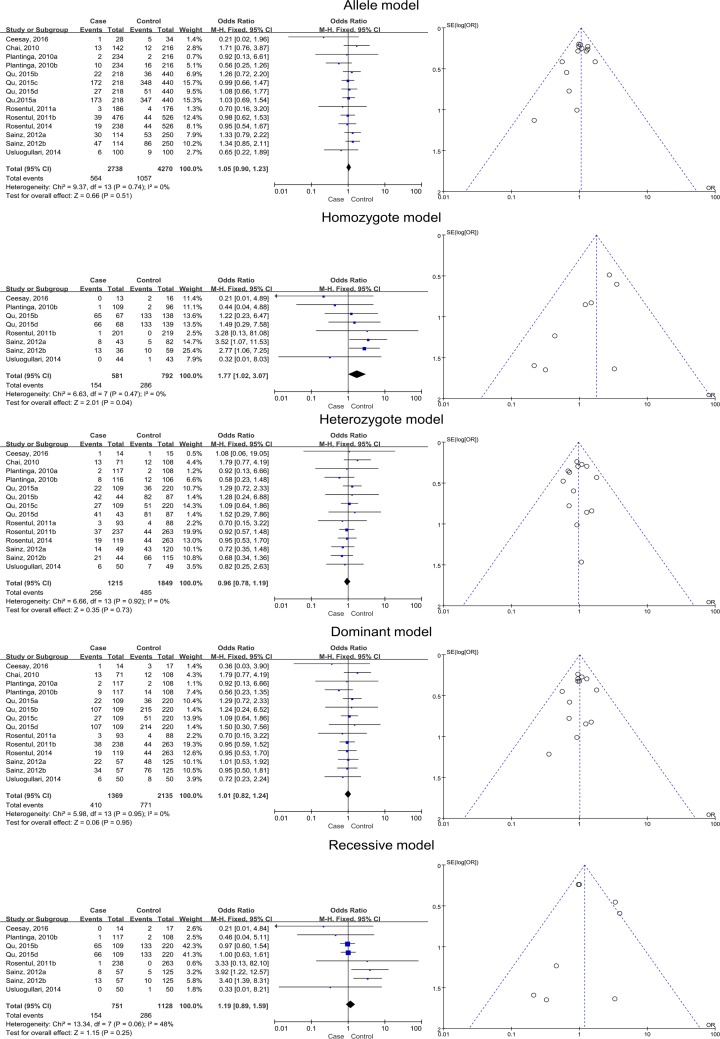
Relationships between dectin-1 gene SNPs and FI in all five models, as assessed using funnel and Forest plots The SNPs include rs17206002, rs3901533, rs11053613, rs3901532, rs7309123, rs16910526, and rs16910527.

Statistically, the distribution of the homozygote model was significant (*BB* versus *AA*; pooled OR 1.77, 95% CI 1.02–3.07, *P*=0.044), implying that the variant genotype *BB* is associated with increased susceptibility to FI. No significant associations were detected for the allele (*B* versus *A*), heterozygote (*AB* versus *AA*), dominant (*BB* + *AB* versus *AA*), and recessive (*BB* versus *AB* + *AA*) models. Detailed results are provided in Table 3 and Figure 2.

**Table 3:** Meta-analysis of the associations between SNPs of the whole dectin-1 gene and FI

FI type	Genotype	Pooled OR	95% CI	*P-*value	χ^2^	*I^2^*	*P*-value (heterogeneity)
Superficial + deep	Allele model (*B vs. A*)	1.05	0.90–1.23	0.51	9.37	0.0%	0.74
	Homozygote model (*BB vs. AA*)	**1.77**	**1.02–3.07**	**0.04**	6.63	0.0%	0.47
	Heterozygote model (*AB vs. AA*)	0.96	0.78–1.19	0.73	6.66	0.0%	0.92
	Dominant model (*BB* + *AB vs. AA*)	1.01	0.82–1.24	0.95	5.98	0.0%	0.95
	Recessive model (*BB vs. AB* + *AA*)	1.19	0.89–1.59	0.25	13.34	48%	0.06
Superficial	Allele model (*B vs. A*)	0.99	0.81–1.20	0.89	3.42	0.0%	0.84
	Homozygote model (*BB vs. AA*)	0.96	0.37–2.46	0.93	1.22	0.0%	0.75
	Heterozygote model (*AB vs. AA*)	1.03	0.77–1.37	0.86	2.58	0.0%	0.92
	Dominant model (*BB* + *AB vs. AA*)	1.01	0.76–1.33	0.97	3.17	0.0%	0.87
	Recessive model (*BB vs. AB* + *AA*)	0.96	0.69–1.33	0.79	0.83	0.0%	0.84
Deep	Allele model (*B vs. A*)	1.17	0.91–1.51	0.21	4.70	0.0%	0.45
	Homozygote model (*BB vs. AA*)	**2.46**	**1.24–4.86**	**0.01**	2.78	0.0%	0.43
	Heterozygote model (*AB vs. AA*)	0.89	0.66–1.22	0.48	3.67	0.0%	0.60
	Dominant model (*BB* + *AB vs. AA*)	1.01	0.75–1.36	0.96	2.80	0.0%	0.73
	Recessive model (*BB vs. AB* + *AA*)	**2.85**	**1.48–5.47**	**0.002**	3.09	3.0%	0.38

Bold values represent *P*<0.05. Abbreviation: vs., versus.

### Pooled effects for CLEC7A SNPs and different subtypes of FI

**Figure 3:**
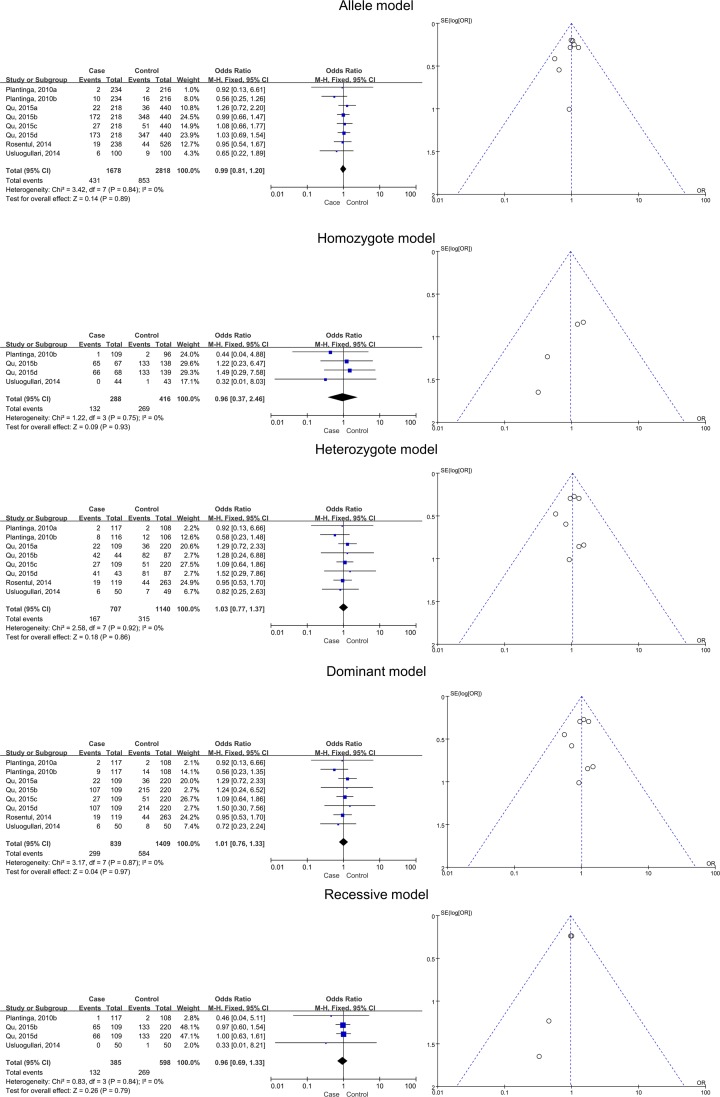
Relationships between dectin-1 gene SNPs and superficial FI in all five models as assessed using funnel and Forest plots The SNPs include rs17206002, rs3901533, rs11053613, rs3901532, rs16910526, and rs16910527.

Four studies with eight subgroups of the superficial type of FI and *CLEC7A* SNPs were subjected to the meta-analysis. After statistical analyses, no significant associations were identified for the allele (*B* versus *A*), homozygote (*BB* versus *AA*), heterozygote (*AB* versus *AA*), dominant (*BB* + *AB* versus *AA*), and recessive (*BB* versus *AB* + *AA*) models (Table 3 and Figure 3).

**Figure 4:**
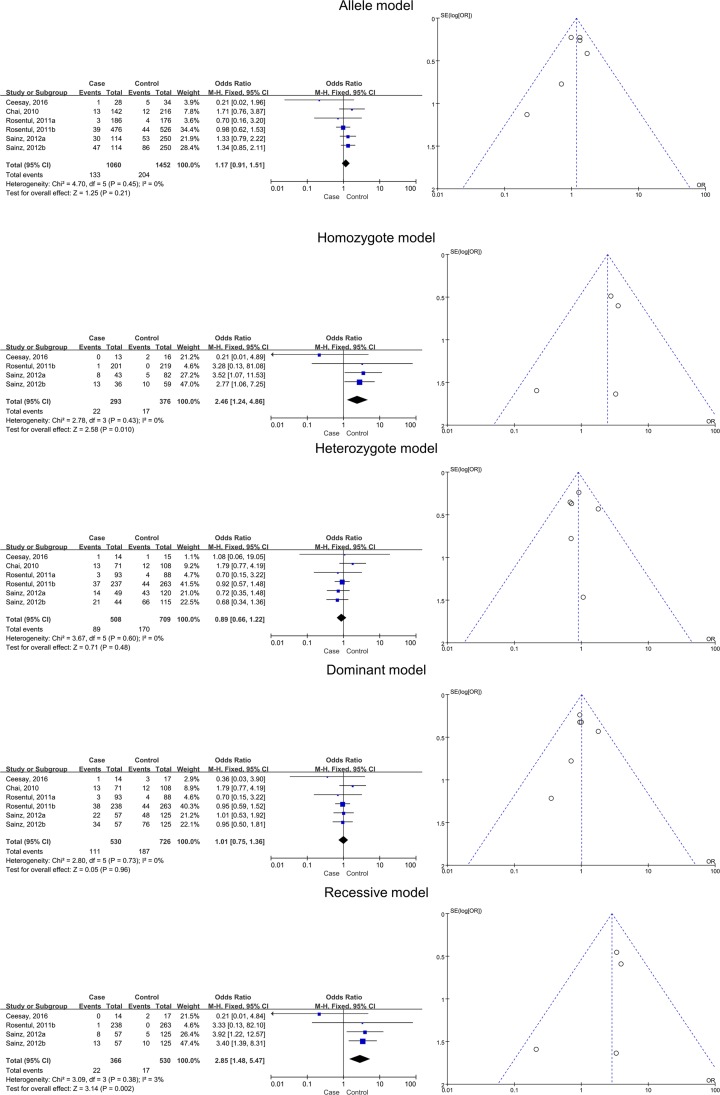
Relationships between dectin-1 gene SNPs and deep FI in all five models as assessed using funnel and Forest plots The SNPs include rs3901533, rs7309123, and rs16910526.

Four studies with six subgroups were subjected to meta-analysis for the deep type of FI. Significant associations between SNPs and increased risk of deep FI risk were detected for the homozygote (*BB* versus *AA*) and recessive (*BB* versus *AB* + *AA*) models (*BB* versus *AA*, pooled OR 2.46, 95% CI 1.24–4.86, *P*=0.01; *BB* versus *AB* + *AA*, pooled OR 2.85, 95% CI 1.48–5.47, *P*=0.002). This suggested that variant allele *B* is associated with susceptibility to deep FI. No significant associations were found for the allele (*B* versus *A*), heterozygote (*AB* versus *AA*), and dominant (*BB* + *AB* versus *AA*) models (Table 3 and Figure 4).

### Pooled effects for CLEC7A rs16910526 SNPs and FI

**Figure 5:**
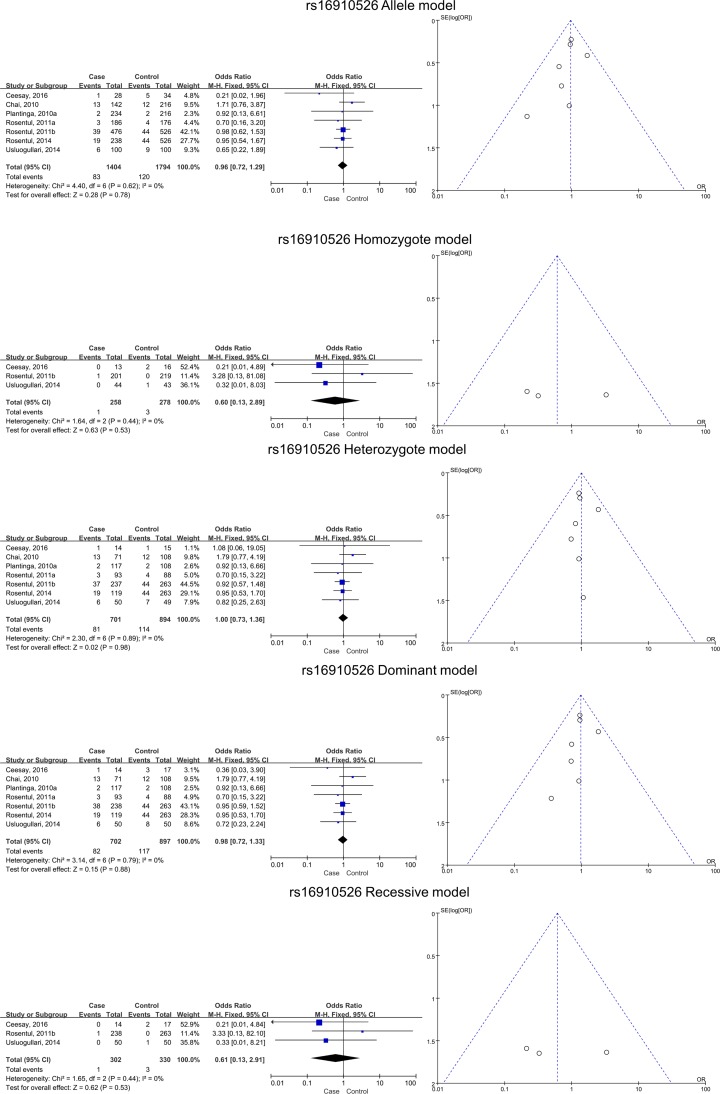
The association between dectin-1 gene SNP rs16910526 and FI in all five models, as assessed using funnel and Forest plots

The data from six included studies provided data associated with seven subgroups. There was no significant association between FI risk and rs16910526 in the allele (*B* versus *A*), homozygote (*BB* versus *AA*), heterozygote (*AB* versus *AA*), dominant (*BB* + *AB* versus *AA*), and recessive (*BB* versus *AB* + *AA*) models, whether analyzed in a whole or in subtypes of superficial and deep FI (Table 4 and Figures 5 and 7).

**Table 4:** Meta-analysis of the associations between SNP rs16910526 and FI

FI type	Genotype	Pooled OR	95% CI	*P-*value	χ^2^	*I^2^*	*P*-value (heterogeneity)
Superficial + deep	Allele model (*B vs. A*)	0.96	0.72–1.29	0.78	4.40	0.0%	0.62
	Homozygote model (*BB vs. AA*)	0.60	0.13–2.89	0.53	1.64	0.0%	0.44
	Heterozygote model (*AB vs. AA*)	1.00	0.73–1.36	0.98	2.30	0.0%	0.89
	Dominant model (*BB* + *AB vs. AA*)	0.98	0.72–1.33	0.88	3.14	0.0%	0.79
	Recessive model (*BB vs. AB* + *AA*)	0.61	0.13–2.91	0.53	1.65	0.0%	0.44
Superficial	Allele model (*B vs. A*)	0.88	0.54–1.42	0.59	0.40	0.0%	0.82
	Homozygote model (*BB vs. AA*)	/	/	/	/	/	/
	Heterozygote model (*AB vs. AA*)	0.92	0.55–1.53	0.74	0.05	0.0%	0.98
	Dominant model (*BB* + *AB vs. AA*)	0.89	0.54–1.48	0.66	0.18	0.0%	0.91
	Recessive model (*BB vs. AB* + *AA*)	/	/	/	/	/	/
Deep	Allele model (*B vs. A*)	1.01	0.70–1.47	0.95	3.73	20.0%	0.29
	Homozygote model (*BB vs. AA*)	0.76	0.12–4.76	0.77	1.43	30.0%	0.23
	Heterozygote model (*AB vs. AA*)	1.05	0.70–1.56	0.82	2.09	0.0%	0.55
	Dominant model (*BB* + *AB vs. AA*)	1.03	0.70–1.52	0.88	2.76	0.0%	0.43
	Recessive model (*BB vs. AB* + *AA*)	0.76	0.12–4.76	0.77	1.43	30.0%	0.23

Abbreviation: vs., versus.

### Evaluation of heterogeneity and publication bias

No significant heterogeneity among the studies was found in the meta-analysis (the *I^2^* values were all less than 50% and all *P*-values of heterogeneity were greater than 0.05). In the meta-analysis, the *I^2^* value for the association between the *CLEC7A* SNPs and FI was 48% in the recessive model (*BB versus AB* + *AA*); therefore, Begg’s test (*P*=0.902) and Egger’s test (*P*=0.830) were used to confirm the result. RevMan 5.3 was used to estimate the publication bias for the meta-analyses, and the results were exported in the form of funnel plots (Figures 2 and 7).

**Figure 6:**
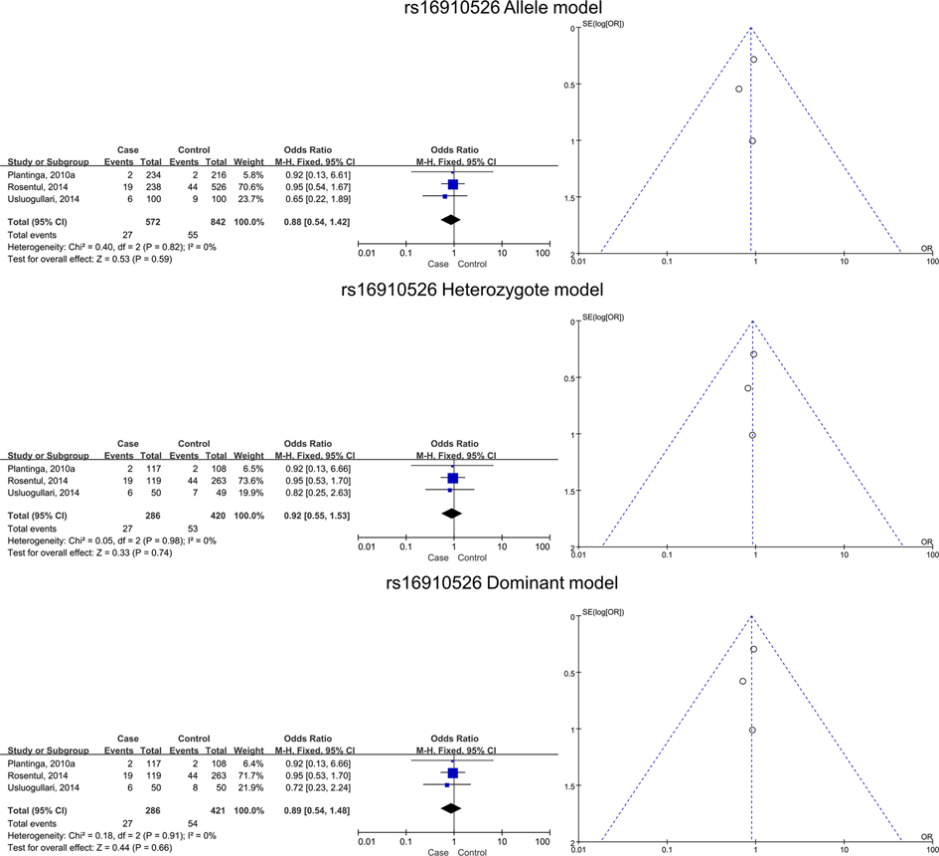
The association between dectin-1 gene SNP rs16910526 and superficial FI in all five models, as assessed using funnel and Forest plots

**Figure 7:**
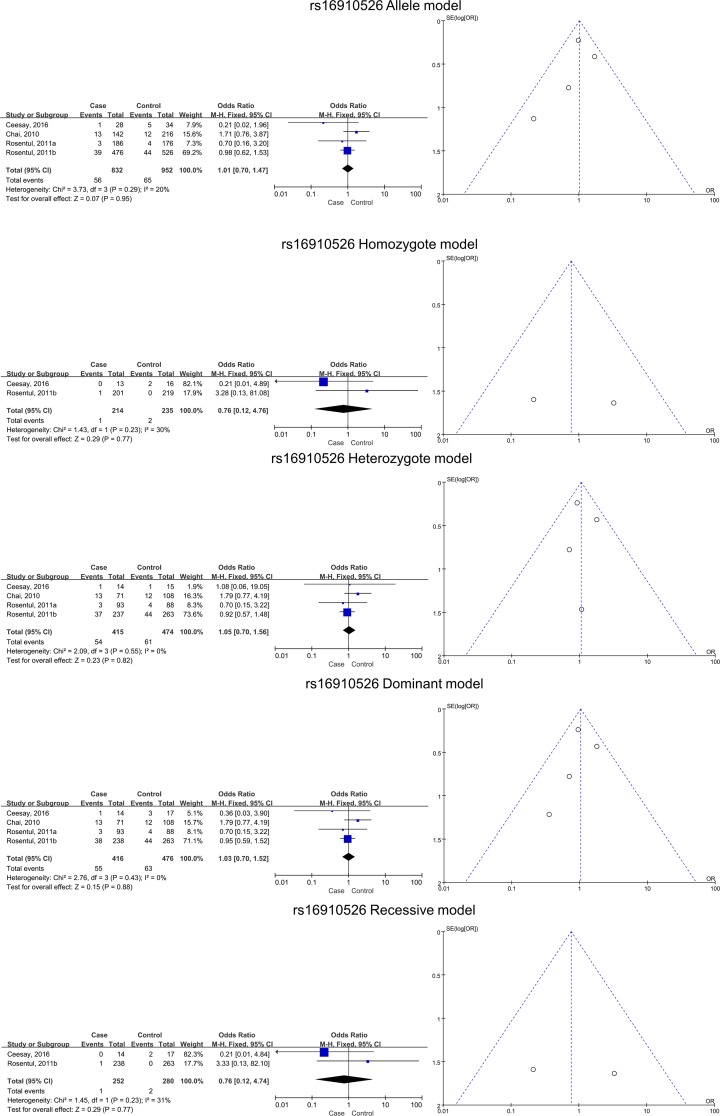
The association between dectin-1 gene SNP rs16910526 and deep FI in all five models, as assessed using funnel and Forest plots

## Discussion

Epidemiological studies have demonstrated that combinations of predisposing factors, including genetic factors, affect the risk of developing FI [21]. Dectin-1, a PRR, can initially recognize fungal pathogens via pathogen-associated molecular patterns (PAMPs) present in the cell walls of fungi. Thereafter, a signaling cascade is initiated, resulting in cytokines and chemokines production. Neutrophil recruitment is stimulated by these cytokines and chemokines, resulting in antigen-specific immunity. Therefore, dectin-1 has a crucial function in fungal pathogen defense initiation [22]. Gene polymorphisms have been frequently associated with the pathology of FI. By systematically analyzing the association between SNPs in *CLEC7A* and the risk of FI, we found that *CLEC7A* polymorphisms significantly increased the susceptibility to FI in the homozygote model (*BB vs. AA*). Moreover, when we divided FI into deep and superficial subtypes, a similar increasing trend was detected for the deep FI subtype in both the homozygote and recessive models (*BB vs. AA* and *BB vs. AB* + *AA*). However, no significant association was detected for the superficial subtype in any model. Thus, possessing the *CLEC7A* variant allele *B* and maintaining the homozygous *BB* genotype might increase the host’s susceptibility to deep FI.

The present review assessed the association between FI susceptibility and *CLEC7A* SNPs, including rs17206002, rs3901533, rs11053613, rs3901532, rs7309123, rs16910526, and rs16910527. A previous study showed that carrying the *CLEC7A* rs3901533 and rs7309123 *BB* genotypes is associated with a significantly increased risk of invasive pulmonary aspergillosis infection [10]. Moreover, another included study [16] demonstrated that SNP rs16910527 might increase susceptibility to oropharyngeal candidiasis in HIV-positive patients. However, the associations between rs16910526 and FI were analyzed using a meta-analysis independently, and no significant association was found, either for overall FI or for the deep and superficial subtypes. Additionally, no significant association was demonstrated between *CLEC7A* polymorphisms (rs17206002, rs3901533, rs11053613, rs3901532) and fungal keratitis risk [14]. Overall, although some studies have demonstrated an association between FI and *CLEC7A* SNPs, there is some inconsistency; therefore, additional case–control, cohort, and other types of study are required to further validate and extend these results.

Several studies other than case–control studies have analyzed the association between *CLEC7A* SNPs and FI. For example, Cunha et al. [23] found a high risk of aspergillosis when SNP rs16910526 was present simultaneously in both recipients and donors during hematopoietic stem cell transplantation (HSCT). Furthermore, Plantinga et al. [24] demonstrated that patients undergoing HSCT who carried the rs16910526 SNP in *CLEC7A* were more susceptible to *Candida* species colonization than patients bearing wild-type *CLEC7A* (OR 11.9, 95% CI 2.5–56.8). Similar results were obtained by van der Velden et al. [25], who showed that *Candida* spp. colonization was more frequent in patients who had undergone allogeneic HSCT and who carried the loss-of-function SNPs rs16910526, compared with that in patients carrying the wild-type allele (73 versus 31%; *P*=0.002). By contrast, the results of Fischer et al. [26] did not demonstrate a correlation between carrying SNP rs16910526 and pulmonary invasive fungal disease (IFD) in patients diagnosed with acute myelocytic leukemia (AML) (OR 0.7; 95% CI 0.2–2.5, *P*=0.65). However, patients carrying the *CLEC7A* rs7309123 *BB* or (*BB* and *AB*) genotype had a significantly higher risk of developing pulmonary IFD (OR 2.6, *P*=0.012; OR 2.4, *P*=0.041; respectively) [26]. Another study demonstrated that the presence of SNP rs16910526 in HIV-positive patients did not influence the incidence of oropharyngeal candidiasis or other opportunistic infections [27]. These previous results are basically in accordance with our study as no significant association between rs16910526 and FI was found by meta-analysis.

The present study had several limitations. Language barriers meant that only publications written in English and Chinese were included. This may have resulted in selection bias of the studies. Thus, further updated meta-analyses involving any studies that are written in other languages are required. Additionally, the different SNPs in *CLEC7A* were analyzed as a whole, and only rs16910526 SNP was analyzed individually; the other SNPs were not subjected to meta-analyses because of the limited number of eligible studies. To avoid possible inconsistency in the results, more studies to determine the association between FI and different *CLEC7A* SNPs are needed. Notwithstanding these limitations, the present study contributes to a deeper understanding of the association between *CLEC7A* SNPs and FI. However, the results should be considered with caution.

## Conclusion

The pathophysiology of FI is supposed to be associated with genetic polymorphisms. This systemic review suggested that *CLEC7A* SNPs rs3901533, rs7309123, and rs16910527 might play a role in FI susceptibility. The meta-analysis provided further evidence that *CLEC7A* SNPs might influence susceptibility to deep FI. However, rs16910526 polymorphisms may have no significant effect. These data demonstrated the critical role of dectin-1 genetic variations in FI. Further investigations are warranted to verify and extend the present results to design novel immunotherapeutic strategies to optimize or replace conventional antifungal treatments.

## Abbreviations

CI: confidence interval
CLEC7A: C-type lectin domain family 7 member A
FI: fungal infection
HSCT: hematopoietic stem cell transplantation
IFD: invasive fungal disease
ITAM: immunoreceptor tyrosine-based activation motif
OR: odds ratio
PRR: pattern recognition receptor
RevMan: Review Manager
SNP: single nucleotide polymorphism
